# Defining electronic-prescribing and infusion-related medication errors in paediatric intensive care – a Delphi study

**DOI:** 10.1186/s12911-018-0713-8

**Published:** 2018-12-07

**Authors:** Moninne M. Howlett, Brian J. Cleary, Cormac V. Breatnach

**Affiliations:** 1 Our Lady’s Children’s Hospital, Crumlin, Dublin, 12 Ireland; 2 School of Pharmacy, Royal College of Surgeons in Ireland, Dublin 2, Ireland; 3 National Children’s Research Centre, Crumlin, Dublin, 12 Ireland; 4 Rotunda Hospital, Dublin, 1 Ireland

**Keywords:** Paediatric intensive care, Medication errors, Patient safety, Health information technology, Medical order entry systems, Infusion pumps, Consensus

## Abstract

**Background:**

The use of health information technology (HIT) to improve patient safety is widely advocated by governmental and safety agencies. Electronic-prescribing and smart-pump technology are examples of HIT medication error reduction strategies. The introduction of new errors on HIT implementation is, however, also recognised. To determine the impact of HIT interventions, clear medication error definitions are required. This study aims to achieve consensus on defining as medication errors a range of either technology-generated, or previously unaddressed infusion-related scenarios, common in the paediatric intensive care setting.

**Methods:**

This study was conducted in a 23-bed paediatric intensive care unit (PICU) of an Irish tertiary paediatric hospital. A modified Delphi technique was employed: previously undefined medication-incidents were identified by retrospective review of voluntary incident reports and clinical pharmacist interventions; a multidisciplinary expert panel scored each incident using a 9-point Likert scale over a number of iterative rounds; levels of agreement were assessed to produce a list of medication errors. Differences in scoring between healthcare professionals were assessed.

**Results:**

Seventeen potential errors or ‘scenarios’ requiring consensus were identified, 13 of which related to technology recently implemented into the PICU. These were presented to a panel of 37 participants, comprising of doctors, nurses and pharmacists. Consensus was reached to define as errors all reported smart-pump scenarios (*n* = 6) and those pertaining to the pre-electronic process of prescribing weight-based paediatric infusions (*n* = 4). Of 7 electronic-prescribing scenarios, 4 were defined as errors, 2 were deemed not to be and consensus could not be achieved for the last. Some differences in scoring between healthcare professionals were found, but were only significant (*p* < 0.05) for two and three scenarios in consensus rounds 1 and 2 respectively.

**Conclusion:**

The list of medication errors produced using the Delphi technique highlights the diversity of previously undefined medication errors in PICU. The increased complexity of electronic-prescribing processes is evident from the difficulty in achieving consensus on those scenarios. Reducing ambiguity in defining medication errors should assist future research on the impact of HIT medication safety initiatives in critical care. The increasing use of HIT and associated new errors will necessitate further similar studies.

## Background

Recent decades have seen two distinct but related healthcare developments; the patient safety movement and the drive for increased use of health information technology (HIT) or eHealth [1–4]. The potential of HIT to reduce medication errors, the most common preventable cause of patient harm, has been identified in numerous safety agency and governmental reports [5–7]. Paediatric patients are at increased risk from medication error, with the paediatric and neonatal intensive care setting being of particular concern [8, 9]. The complexity of illness, the use of multiple high-risk medications and the vast range in weights of patients cared for are significant factors [10, 11]. As a result, there has been an influx of technology based interventions into this environment, aimed at mitigating these inherent risks [12, 13]. Electronic-prescribing, more commonly referred to as computerised provider order entry (CPOE) in the US, and the use of smart-pump technology are primary examples [14–16].

Paediatric-specific HIT systems do not however exist, and the current evidence for the effectiveness of HIT in preventing paediatric medication errors is limited [15, 17–19]. The specific functionalities required for the paediatric setting have been highlighted [20, 21]. The considerable financial cost and extensive process changes that HIT brings to healthcare warrant robust evaluation and rigorous research. Additionally, the introduction of new errors on implementation of HIT is a widely reported phenomenon [22–24]. Capturing the impact of these well intended initiatives is difficult [12]. One impediment is the absence of suitably specific error definitions [22, 25, 26]. The ‘niche’ nature of paediatrics, and the fact that many existing definitions pre-date HIT implementation, are particular barriers [27, 28]. Heterogeneity of medication use processes, with differing levels of HIT integration, and local customisation of both home-grown and commercially available systems, are further impediments [23, 29–31].

A germane example of both the ‘niche’ component and the need for clearer definitions is the current movement in both Europe and the US to standardise paediatric and neonatal infusions [32–34]. The use of traditional individualised weight-based infusions is recognised as being error-prone, yet remains common practice in many paper-based neonatal and paediatric settings across Europe [33, 35, 36]. Furthermore, widespread implementation of smart-pumps has yet to happen, with many units continuing to administer high-risk medications via traditional infusion pumps [33, 34, 36]. Current definitions fail to adequately catch the types of errors associated with these contrasting infusion processes.

The Delphi technique is a methodology used in health research to achieve consensus among groups of experts on particular issues where none exists. It consists of multiple iterative rounds, involving an expert panel, who are provided with controlled anonymous feedback [37, 38]. It has previously been used, in the pre-HIT era, to produce practitioner-based definitions of both paediatric and adult prescribing errors, with accompanying lists of included medication incidents [27, 39].

The aim of this study was to achieve consensus in defining a range of new technology-generated scenarios related to electronic prescribing, the use of smart-pump technology, and the interface between these two HIT interventions. To facilitate this work, it was also necessary to seek consensus on a range of previously unaddressed weight-based infusion scenarios still common in paediatric and neonatal intensive care settings. We aim to mirror the process used in earlier similar studies, thereby enabling the results of this study to be used in conjunction with them.

A secondary aim is to identify any significant differences between the specific healthcare professional (HCP) groups in the expert panel. The medication use process is complex, involving multiple stages from point of medication ordering to patient administration, and is reliant on a range of HCPs. Successful HIT implementation requires an understanding of these different roles and their points of interaction with each HIT system [23]. In an era of rapid change in the use of HIT we hope, that by systematically defining novel medication errors, this study will assist future research on the impact of HIT medication safety initiatives.

## Methods

### Setting

**Fig. 1:**
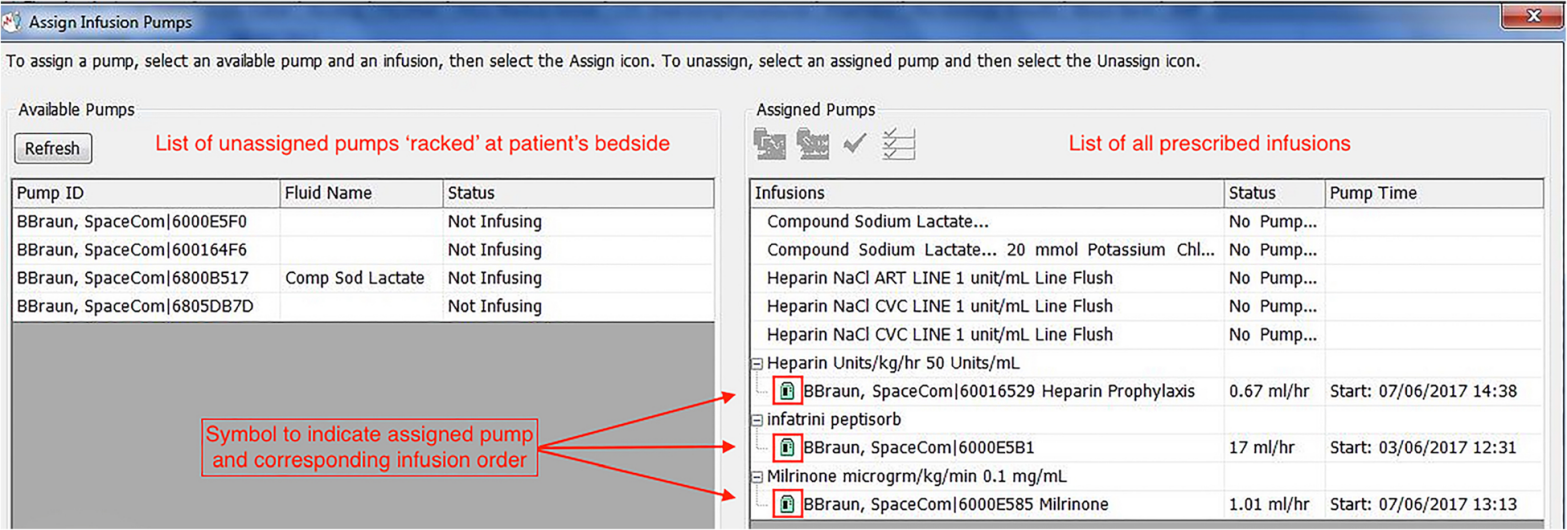
Screenshot of Manual Assignment of Infusion Pump to Corresponding Electronic Infusion Order

This study was conducted in a 23-bed Paediatric Intensive Care Unit (PICU) in a tertiary care paediatric hospital in Dublin, Ireland. In 2012, a clinical information management system (Phillips ICCA®) was implemented, which facilitated electronic medication ordering and medication administration records. Secondly, traditional weight-based infusions were replaced with standard concentration infusions (SCIs). An agreed drug library of SCIs was uploaded onto B.Braun Space® infusion pumps. A uni-directional interface was established between the electronic infusion orders and the pumps such that real-time infusion data is transferred directly to the clinical information management system. This interface is reliant on manual assignment by nursing staff of the infusion pump to the corresponding infusion order. Images of this process can be seen in Fig. 1. Barcode assisted medication administration tools were not in use.

### Delphi consensus process

A multi-phase modified Delphi technique was employed to achieve consensus on a range of previously unaddressed medication scenarios across three different categories.

### Stage 1: Identification of scenarios

A modified Delphi technique was used, whereby the research team identified the scenarios requiring consensus. This differs from the traditional Delphi method, where the panel of experts is involved in this initial phase of the process [37]. Recorded incidents covering a 4-year period before and after the implementation of the new technology were analysed to identify those incidents not amenable to categorisation as ‘errors’ using previously published lists. Incidents from both the PICU clinical pharmacists’ intervention records and the hospital’s voluntary medication incident reporting system were included. Incidents directly attributable to the new technology were identified, and further categorised as being either electronic-prescribing or smart-pump technology related. A third category consisted of incidents related to the pre-electronic prescribing of weight-based infusions.

### Stage 2: Selection of the expert panel

The highly specialised nature of the PICU setting necessitated a mixture of convenience and purposive sampling to recruit suitably experienced participants. Participation by the panel was on a voluntary basis, with no incentives offered or provided. All individuals meeting the following criteria were personally invited to participate:

- Paediatric Intensive Care consultants and registrars
- Senior PICU nursing staff rostered the day of the initial round of consensus. Participating nurses must have completed a Graduate Diploma in Paediatric Critical Care Nursing
- Pharmacists with either direct experience of the current medication process in our PICU, or that of another PICU
- Clinical pharmacists, of at least senior pharmacist grade (> 3 years’ experience), without PICU experience but with good knowledge and understanding of paediatric medication errors. This group was provided with supplementary demonstrations of each scenario on the clinical information management system where necessary

### Stage 3: Iterative consensus rounds

A combination of paper-based and e-Delphi techniques were employed during the iterative consensus rounds. After each round, controlled feedback, comprising of each individual’s response accompanied by the distribution of the group’s response, was provided [37, 40, 41]. Participants were instructed that they need not conform to the group view.

#### Round 1

All participants attended a presentation, which was repeated 3 times over the course of one day. They were presented with a brief description of each scenario, and a corresponding image or screen shot from the clinical information management system where appropriate. Where a similar but non-corresponding scenario from previously defined lists in the paediatric [27], or adult [39], settings existed, this scenario and its outcome from those studies was also presented to the group. A specific medication error definition was not provided.

Each participant independently scored each scenario using a 9-point Likert scale. A score of 1 indicated “definitely not an error” and a score of 9 indicated “definitely an error”. Participants were also invited to record comments on individual scenarios.

The median and inter-quartile range (IQR) for each scenario was calculated using Microsoft Excel® and the results analysed for degree of consensus. The following pre-determined consensus definitions were applied:

1. ‘Consensus’ was considered to exist if the interquartile range of the participants’ responses fell within any three-point range
2. ‘Disagreement’ existed if the interquartile range spanned both the 1–3 range and the 7–9 range
3. If neither consensus nor disagreement existed, ‘Partial Agreement’ was considered to have occurred

Where consensus existed, it was considered that the scenario would be included as an error if the median score fell within the 7–9 range, excluded if it fell within the 1–3 range, and would be considered equivocal if it fell within the 4–6 range.

#### Round 2

Those scenarios for which consensus was not reached were included in Round 2. This was conducted by e-mail in consideration of the complex rostering of PICU staff. Each participant was sent the Round 2 scenarios, with that individual’s corresponding Round 1 score, the median and inter-quartile range (IQR) scores for the entire group and any comments from Round 1. Participants were asked to resubmit their scores, with the option to amend or retain their Round 1 score having considered the group results.

#### Round 3

A third round was deemed necessary for two complex electronic-prescribing scenarios. A short video was prepared for each scenario which demonstrated both the process and outcome of that scenario on the electronic-prescribing system. To reduce the demands being made on the participants, each participant was sent a brief online survey (SurveyMonkey Inc. Palo Alto, California, USA), with a link to the two videos.

### Statistical analysis

Using Stata 13.1, any differences between healthcare professions (HCPs) in the scoring of scenarios were identified using Chi-square tests. Symmetry and marginal homogeneity tests were also run to examine the effect of the iterative process during successive rounds on the scores for each scenario. Statistical significance was defined to a *p*-value of less than or equal to 0.05. No adjustments were made for multiple comparisons.

## Results

### Delphi stage 1 (identification of scenarios)

Thirteen technology-generated scenarios were identified, seven pertaining to electronic-prescribing, and six to smart-pump technology. Four scenarios pertaining to the pre-electronic process of prescribing weight-based paediatric infusions were also identified. See Table 1. All the electronic-prescribing and paper-based infusion scenarios related to the prescribing phase of the medication use process. The six smart-pump technology scenarios pertain to the introduction of smart-pump technology as a stand-alone intervention or due to the interface between the pumps and the clinical management information system.

**Table 1:** List of Scenarios Identified in Stage 1 and Results from Stage 3

Category 1: Electronic-prescribing (Technology Generated)						
	Scenario	Scores (Median, IQR^a^)			Outcome	
		Rd^c^ 1	Rd 2	Rd 3	Consensus	Error
1	Alteration of a standard order from a dropdown menu resulting in incongruous supplementary instructions	7 (4)	7 (1.5)	x	✓	✓
2	Alteration of an existing order resulting in incorrect supplementary instructions	7 (4)	7 (2)	x	Partial	✓
3	Selection of an incorrect formulation (caused by failure to amend default formulation)	7 (4)	7 (4)	7 (1.8)	✓	✓
4	Inappropriate completion of ‘Max Dose’ field (removing autofilled dose on MAR^d^, causing potential to administer doses outside dose/weight limits)	6 (5)	6 (4)	6 (3.8)	Partial	Equivocal
5	Medication (Infusion Order) not cancelled 48–72 h after *written* and not used e.g. Inotropes on Order Set	4 (5)	4 (2)	x	Partial	✕
6	Medication (Infusion Order) not cancelled 48–72 h after infusion *discontinued*	4 (5)	4 (4)	x	Partial	✕
7	Prescription duplication	8 (3)	8 (2)	x	✓	✓
Category 2: SMART-PUMPS (Technology Generated)						
	Scenario	Scores (Median, IQR^a^)			Outcome	
		Rd^c^ 1	Rd 2	Rd 3	Consensus	Error
8	Wrong patient weight programmed	9 (0)	x	x	✓	✓
9	Wrong drug programmed from drug library	9 (0)	x	x	✓	✓
10	Wrong rate programmed	9 (0)	x	x	✓	✓
11	Incorrect SCI^b^ programmed (not equal to SCI ordered or prepared) – incorrect dose administered	9 (0)	x	x	✓	✓
12	Incorrect SCI prepared (pump programmed with SCI ordered rather than SCI prepared)-incorrect dose administered	9 (0)	x	x	✓	✓
13	Programmed as per SCI in syringe but pump assigned to incorrect electronic infusion order (resulting in incorrect auto-charting of dose administered)	9 (1)	x	x	✓	✓
Category 3: Prescribing of PICU Infusions						
	Scenario	Scores (Median, IQR^a^)			Outcome	
		Rd^c^ 1	Rd 2	Rd 3	Consensus	Error
14	Ordering an infusion in the wrong concentration for a patient without valid clinical rationale	9 (1)	x	x	✓	✓
15	Writing an incorrect statement of rate (*type A*): expressing rate as X ml (rather than Xml/hour) = dose/weight/time	8 (2)	x	x	✓	✓
16	Writing an incorrect statement of rate (*type B*): expressing rate using incorrect unit of time e.g. ‘per min’ instead of ‘per hour’	9 (1)	x	x	✓	✓
17	Writing an incorrect statement of rate: Combination of both *type A* and *type B* error	9 (1)	x	x	✓	✓

^a^*IQR* Interquartile Range, ^b^*SCI* Standard Concentration Infusion, ^c^*Rd* Consensus Round, ^d^*MAR* Medication Administration Record

### Delphi stage 2 (selection of the expert panel)

A total of 37 participants participated in Round 1. This included 15 doctors, 13 nurses and 9 pharmacists. All doctors and nurses, and 7 of the 9 pharmacists were employed in our institution.

### Delphi stage 3 (iterative consensus rounds)

**Fig. 2:**
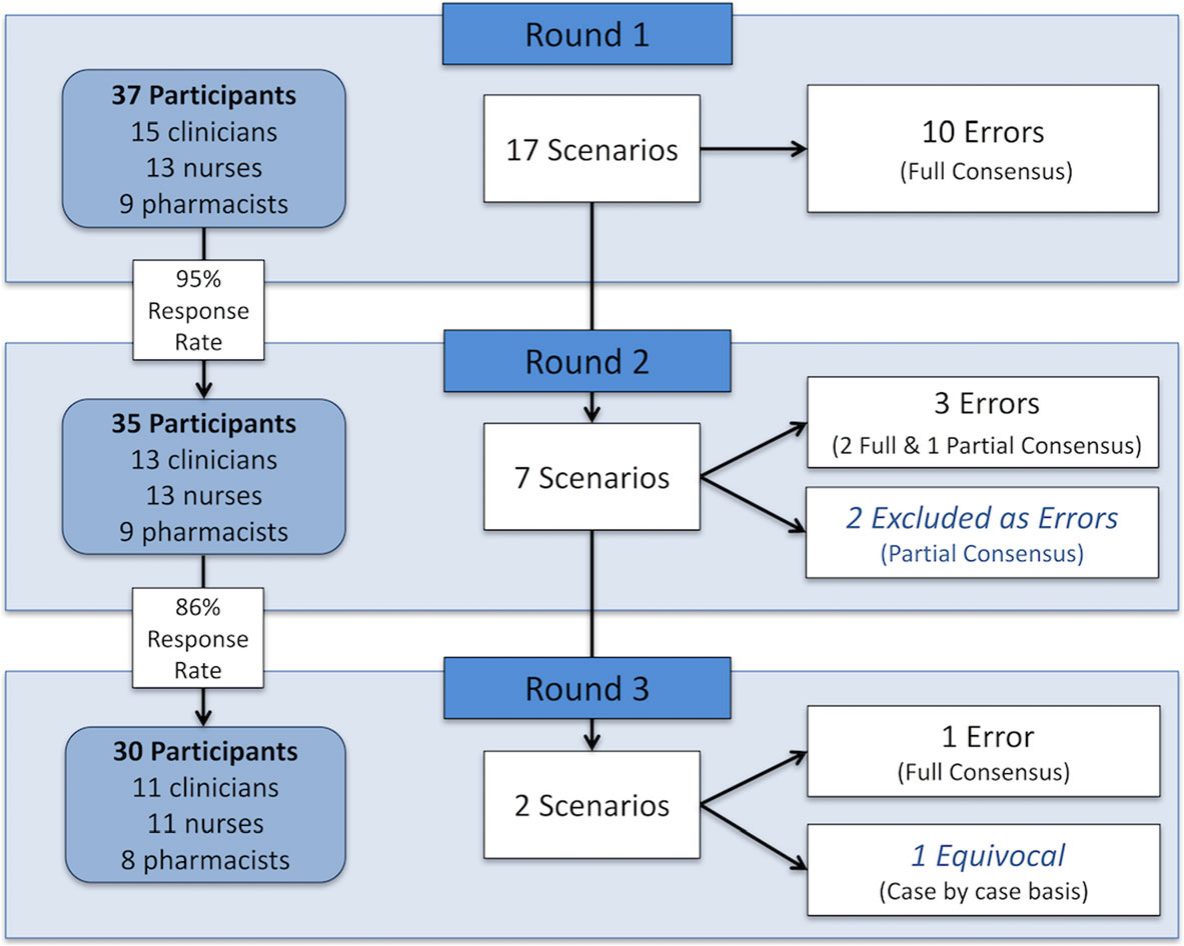
Overview of Consensus Process

Round 1 produced consensus to define 10 of the 17 scenarios as errors. This included all smart-pump and paper-based prescribing of PICU infusion scenarios. All electronic-prescribing scenarios (*n* = 7) required progression to Round 2, where consensus to define two further incident types as errors was reached. Partial consensus was reached on three of the remaining five. Based on feedback comments from Round 1 or any significant changes in scores identified by statistical analysis, it was decided to include one and exclude two of these as errors. Round 3 produced a consensus to include one of the final two scenarios as an error, with no further agreement being attained on the second. It was decided that this scenario remained equivocal and would therefore need to be judged on a case-by-case basis. See Fig. 2.

Response rates were 95% (*n* = 35) and 86% (*n* =  30) in Rounds 2 and 3 respectively. Any missing scores identified were clarified with the respondents such that all respondents scored all scenarios.

**Fig. 3:**
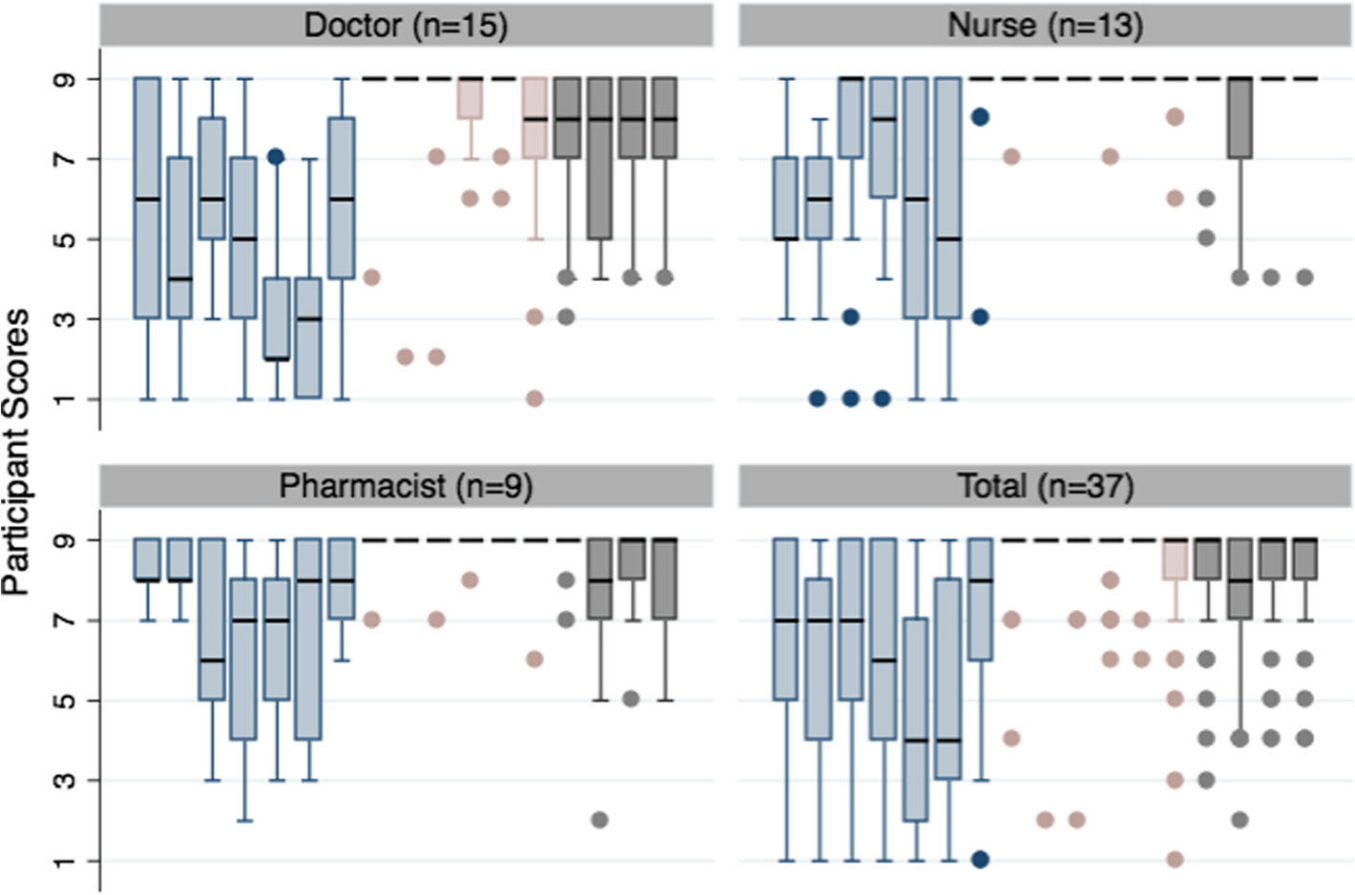
Boxplots of Median/Interquartile Range Scores for Scenarios in Round 1 (by Healthcare Profession)

Variability in scoring is highest across all professions for the electronic-prescribing scenarios. Pharmacists appeared to provide higher scores than doctors or nurses, but Chi-square tests indicate this is only significant for scenarios 2 and 5 (*p* = 0.045 and 0.037). See Fig. 3.

**Fig. 4:**
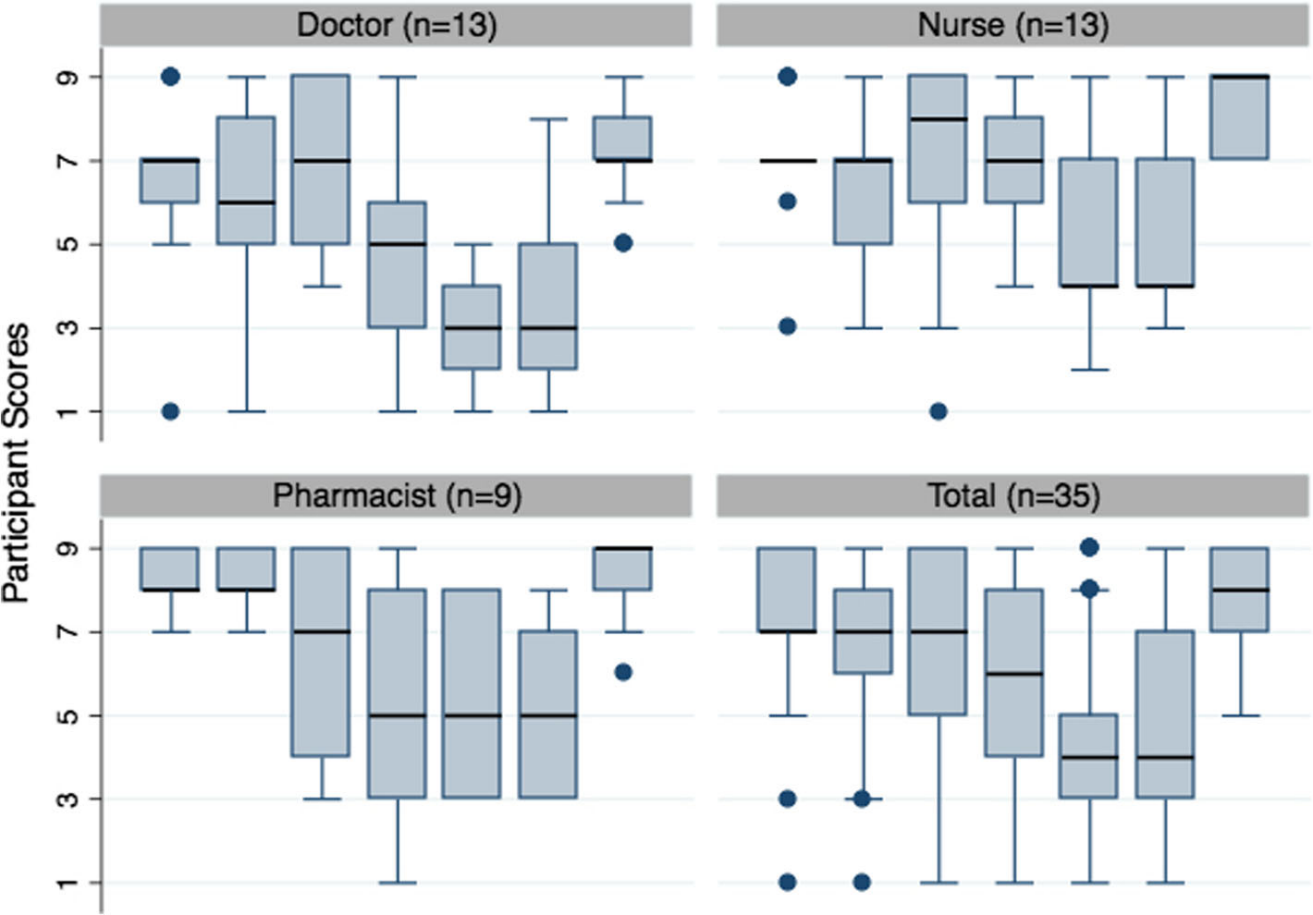
Boxplots of Median/Interquartile Range Scores for Scenarios in Round 2 (by Healthcare Profession)

Although higher levels of consensus were achieved in Round 2, with full or partial consensus being reached on 5 of the 7 scenarios, differences between HCPs were more pronounced. Differences between HCPs were significant for scenarios 1,5 and 6 (*p* = 0.010, 0.009 and *p* = 0.042). Again, this is likely to be due to higher scores from the pharmacists. See Fig. 4. No significant differences were found between HCPs in Round 3.

Results of symmetry and marginal homogeneity tests indicate that the iterative process during successive rounds had little effect on the participants’ scores. Despite increasing levels of consensus, only one scenario showed significance in changing of scores between Round 1 and Round 2 (*p* = 0.0312). A significant lowering of scores for Scenario 5 was found, with half as many 9 scores, and twice as many 4 and 5 scores recorded. Hence this scenario: failure to discontinue infusion orders that had never been used was excluded as an error after Round 2. No significant differences in scores were found in Round 3, despite consensus being reached on scenario 3.

## Discussion

This study demonstrates that achieving consensus for different categories of HIT-related medication errors varies considerably depending on the particular associated HIT system. The complexity of electronic-prescribing systems is evident in the diversity of individual opinion on the seven included scenarios. All were included in Round 2, with two requiring further clarification in Round 3. This difficulty in obtaining consensus is indicative of the nuances in such complex HIT systems, particularly in the paediatric setting.

**Fig. 5:**
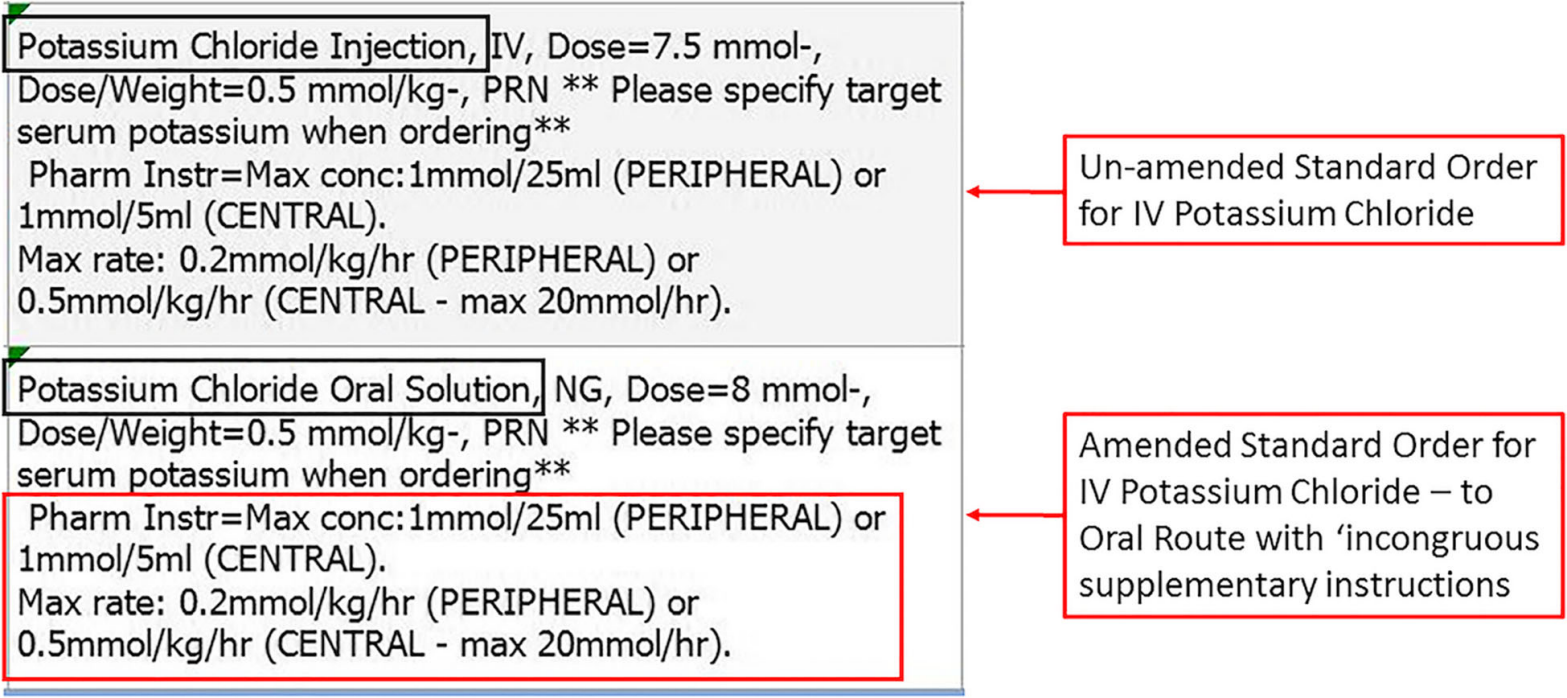
Example of Scenario 1: *Alteration of a standard order from a dropdown menu resulting in incongruous supplementary instructions*

Scenarios 1–4 in this study are examples of the difficulty in customising a commercial system to facilitate the prescribing of a large range of medications to a diverse population. An example of Scenario 1 can be seen in Fig. 5.

Configuring robust dose range checking and clinical decision support in the paediatric setting is particularly challenging [42]; paediatric-specific electronic-prescribing or CPOE systems do not exist [18]. Scenarios 5–7, which involve failure to discontinue orders and duplication, are examples of errors commonly reported with implementation of CPOE [43, 44]. Although they can also occur with paper-based systems and similar scenarios are listed in the UK adult and paediatric practitioner-based studies, these do not capture the complexity of electronic-prescribing. When an electronic order is not discontinued, it stays ‘live’ for the patient’s full length of stay. This differs considerably from paper systems where the paper prescription and administration record becomes full after a set number of days and needs to be rewritten. Also, when an electronic order is altered on our system, a new order is automatically generated. In addition to the risks associated with certain fields inappropriately auto-filling as identified in scenario 2, the number of similar/duplicate orders is also increased.

The consensus position reached in this study was that failure to discontinue orders should not be considered a medication error. The participants considered that this did not pose a risk to the patient and in the dynamic intensive care setting may be clinically warranted. In contrast, it was determined that duplicate orders should be categorised as medication errors. This widely reported consequence of electronic-prescribing has the potential to lead to missed or extra doses due to a cluttered medication record. Ability to view medication orders from various screens compounds this risk. In addition, the existence of duplicate orders in our particular clinical information management system can increase the risk of incorrect manual assignment of infusion pumps to corresponding infusion orders. See Fig. 1.

The decision to provide a video demonstration of scenarios for Round 3 did not produce significantly higher levels of consensus. Despite this, feedback from participants indicated that the videos improved their understanding of the scenarios. We propose that this method may be useful in future studies.

Almost unanimous levels of agreement were found for the smart-pump related scenarios. This may be indicative of a widespread appreciation of both the high-risk nature of the commonly used infusions in PICU, and the risks associated with incorrect infusion pump programming. The programming errors identified in scenarios 8–10 are examples of new errors introduced by the use of smart-pump drug libraries. Scenarios 11–13, involving misalignment of standard concentration infusion (SCI) orders, syringes and pumps are also novel. The range of SCIs required to accommodate the complexity and diversity of the paediatric setting makes these particularly problematic. These errors highlight the need for progress towards closed-loop medication processes, however pre-prepared and barcoded infusion solutions remain rare in many European paediatric hospitals. With national standardisation of paediatric infusions underway in Ireland, and being actively pursued in the UK and the USA, clarity on defining these smart-pump related medication errors is important in assessing the limitations of current processes [32–34].

This study also produced consensus on the pre-electronic prescribing of weight-based paediatric infusion scenarios identified. Despite widespread recommendations against it, this practice is still common in Europe and continues to be recommended in paediatric reference books [32, 35, 45]. Scenarios 15–17, highlight common errors that occur when writing the ‘statement of rate’, a necessary component of the prescription to direct the setting of the pump. Although unlikely to cause harm they should not be overlooked, as the potential for harm is considerable should the statement of rate be misinterpreted by nursing staff unfamiliar with a particular infusion [46, 47]. With widespread discrepancies in medication error terminology, clarity on inclusion of these as prescribing errors may assist future studies and support the move away from this commonly used, yet error-prone method.

No single mitigation strategy is likely to avert the range of errors highlighted in this study. Future research is necessary to assess the impact of standard orders and order sets on electronic-prescribing errors. There are currently limited data on their impact on the efficiency or accuracy of the ordering process [48–50]. There are some data to indicate that they increase duplicate error rates [51]. This information is essential to ensure the on-going safe design of electronic ordering systems. The limitation of smart-pumps as a single intervention to prevent IV administration errors is well recognised. Until closed loop medication processes and bar-coded ready-to-use paediatric infusions are readily available in European hospitals, research into the effectiveness of more immediately available solutions, such as 2D barcode labelling, to reduce programming errors, is warranted.

### Limitations

This study has a number of limitations. Some of the electronic-prescribing scenarios may be specific to this particular clinical information management system (Phillips ICCA®). There is considerable diversity in hospital electronic-prescribing systems, and the extensive use of locally configured clinical decision support is likely to produce an ever-increasing range of novel medication errors [14, 52]. However, the Philips ICCA® system is employed widely in over 60 adult and paediatric ICUs in Ireland and the UK alone, (Personal Communication, Philips – October 2016) and these scenarios are therefore, likely to be applicable to those hospitals.

Similarly, the smart-pump scenario created by the manual assignment process may not correlate to other integration processes; however, all other smart-pump scenarios are likely to occur in the many institutions yet to implement pump integration.

The study is limited in the scope of included scenarios, with two of the three categories related only to prescribing errors. At the time of this study, continuous quality improvement, or CQI data reports from the smart-pumps were not available to us. Hence, many of the other administration errors associated with smart-pumps could not be included. Examples of these include by-passing the drug library and identification of limit override attempts. Further studies investigating other stages of the medication use process will be required to extend and further define HIT generated medication errors.

All nurses and doctors, and seven of the nine pharmacists were from the same institution. A broader panel, albeit less familiar with the HIT scenarios, may have produced different results. The principal researcher created and delivered the presentations, and we recognise the potential this had for introducing bias.

## Conclusion

Implementation of health technology systems introduces a range of medication errors not easily categorised using previously published medication error lists. The Delphi technique can successfully be used to define these novel medication errors and assist in measuring outcomes for HIT quality improvement and patient safety initiatives.

Electronic-prescribing errors may be the most difficult to clearly define, particularly as clinical information systems with higher levels of decision support become more widely employed. Further similar studies are likely to be required as the range of HIT generated medication errors expands across the entire medication use process. The use of video demonstrations may be a useful tool.

The results of this study, in conjunction with previously defined lists of medication errors will add to the quality of future medication error studies in both the paediatric and non-paediatric intensive care setting. This includes sites using electronic-prescribing systems, in particular the many adult and paediatric sites in Ireland and the UK using the Philips ICCA® system, sites using smart-pump technology and most neonatal and paediatric intensive care units.

## Abbreviations

CPOE: Computerised provider order entry
HCP: Healthcare professional IQR: Inter-quartile range
HIT: Health information technology
MAR: Medication Administration Record PICU: Paediatric Intensive Care Unit
SCI: Standard concentration infusion
